# The effect of intravenous interleukin-1 receptor antagonist on inflammatory mediators in cerebrospinal fluid after subarachnoid haemorrhage: a phase II randomised controlled trial

**DOI:** 10.1186/1742-2094-11-1

**Published:** 2014-01-03

**Authors:** Navneet Singh, Stephen J Hopkins, Sharon Hulme, James P Galea, Margaret Hoadley, Andy Vail, Peter J Hutchinson, Samantha Grainger, Nancy J Rothwell, Andrew T King, Pippa J Tyrrell

**Affiliations:** 1 The University of Manchester Stroke and Vascular Centre, Manchester Academic Health Sciences Centre, Clinical Sciences Building, Salford Royal NHS Foundation Trust, Stott Lane, Salford M6 8HD, UK; 2 Faculty of Life Sciences, University of Manchester, Manchester M13 9PT, UK; 3 Centre for Biostatistics, University of Manchester, Manchester Academic Health Sciences Centre, Manchester M13 9PT, UK; 4 Academic Division of Neurosurgery, Department of Clinical Neurosciences, University of Cambridge, Cambridge CB2 2QQ, UK

## Abstract

**Background:**

Interleukin-1 (IL-1) is a key mediator of ischaemic brain injury induced by stroke and subarachnoid haemorrhage (SAH). IL-1 receptor antagonist (IL-1Ra) limits brain injury in experimental stroke and reduces plasma inflammatory mediators associated with poor outcome in ischaemic stroke patients. Intravenous (IV) IL-1Ra crosses the blood–brain barrier (BBB) in patients with SAH, to achieve cerebrospinal fluid (CSF) concentrations that are neuroprotective in rats.

**Methods:**

A small phase II, double-blind, randomised controlled study was carried out across two UK neurosurgical centres with the aim of recruiting 32 patients. Adult patients with aneurysmal SAH, requiring external ventricular drainage (EVD) within 72 hours of ictus, were eligible. Patients were randomised to receive IL-1Ra (500 mg bolus, then a 10 mg/kg/hr infusion for 24 hours) or placebo. Serial samples of CSF and plasma were taken and analysed for inflammatory mediators, with change in CSF IL-6 between 6 and 24 hours as the primary outcome measure.

**Results:**

Six patients received IL-1Ra and seven received placebo. Concentrations of IL-6 in CSF and plasma were reduced by one standard deviation in the IL-1Ra group compared to the placebo group, between 6 and 24 hours, as predicted by the power calculation. This did not reach statistical significance (*P* = 0.08 and *P* = 0.06, respectively), since recruitment did not reach the target figure of 32. No adverse or serious adverse events reported were attributable to IL-1Ra.

**Conclusions:**

IL-1Ra appears safe in SAH patients. The concentration of IL-6 was lowered to the degree expected, in both CSF and plasma for patients treated with IL-1Ra.

## Background

Aneurysmal subarachnoid haemorrhage (SAH) accounts for 5% of all strokes, but 25% of stroke-related mortality [1,2]. Of those who survive the initial bleed, up to one-third may develop delayed cerebral ischaemia (DCI) [3]. The mechanism of DCI remains unclear, but vasospasm and inflammation may be involved [4-6] in its progression to a worse outcome, with nimodipine having only a modest impact [7]. Inflammation is a key process in cerebral ischaemia [8] and is driven by the cytokine interleukin-1 (IL-1). IL-1 upregulates the expression of IL-6, which triggers local inflammation and activation of the systemic acute phase response. IL-6 concentrations are elevated in the cerebrospinal fluid (CSF) of patients after SAH and higher concentrations of CSF IL-6 are seen in patients with worse clinical outcomes [9].

The naturally occurring selective antagonist of IL-1, IL-1 receptor antagonist (IL-1Ra) blocks all known actions of IL-1 and is a promising candidate as a treatment for cerebral ischaemia [10,11]. Intracerebroventricular administration of IL-1Ra protects against diverse forms of experimentally induced brain injury in rodents, including ischaemia [12,13], trauma [14], and perinatal hypoxia [15]. In experimental cerebral ischaemia, IL-1Ra is protective when administered intravenously (IV) [16] or subcutaneously (SC) [17], and when administered up to 3 hours after onset [18]. Intravenously administered IL-1Ra crosses the blood–brain barrier (BBB) and can be found in CSF of SAH patients at concentrations that are effective in limiting brain injury in rodents [16]. In ischaemic stroke patients, IV IL-1Ra reduces peripheral inflammation (plasma IL-6, white cell count, and C-reactive protein, CRP) measured 7 days after stroke [19].

Up to 25% of patients with SAH require temporary external CSF drainage [1] to treat hydrocephalus. This offers a unique opportunity to study changes in brain physiology and pharmacology in patients, where these are reflected in CSF. Using this approach, we have shown that IV IL-1Ra crosses the BBB [16] and that experimentally therapeutic CSF concentrations can be achieved within 45 minutes of IV administration [20].

The aim of this study was to determine whether IV IL-1Ra reduces the concentration of mediators of inflammatory activity in the circulation and CSF.

## Methods

### Patient inclusion criteria and data collection

This was a two-centre (Salford Royal NHS Foundation Trust, Salford, and Addenbrooke’s Hospital, Cambridge, UK), randomised, double-blind, placebo-controlled trial. Ethical and appropriate competent authority approvals were obtained. The inclusion and exclusion criteria are shown in Table 1.

**Table 1:** Eligibility criteria for study patients

**Inclusion criteria**	**Exclusion criteria**
Aged 18 years or above.	Known or suspected infection at the time of consideration for the study.
Patients with confirmed aneurysmal SAH who have had an EVD inserted as part of their clinical care, that is expected to remain *in situ* for more than 48 hours.	Known allergy to *Escherichia coli* or any of the constituents of the study medication as established from the patient themselves, reliable representative, or clinical records.
IL-1Ra or placebo can be administered within 72 hours from ictus.	Previous or existing treatment with IL-1Ra.
Patients are likely to remain resident within the study centre for the next 7 days.	Previous or current treatment with medication suspected of interacting with IL-1Ra, such as TNF-α inhibitors.
Normal renal function (serum creatinine <177 μmol L^-1^).	Known to have participated in a clinical trial of an investigational agent or device in the previous 30 days or for the period determined by the protocol of the study the patient has taken part in.
Willing and able to give informed consent or consent available from a patient’s personal representative (usually the next of kin) for study inclusion including agreement in principle to receive study intervention and undergo all study assessments.	Known pregnancy or breast-feeding.
	Clinically significant concurrent medical condition which, at the CI’s (or designee’s) discretion, could affect the safety, tolerability, or efficacy in this study or would interfere with participation, administration of study treatment, or assessment of outcomes. For example, pre-existing malignancy.
	Previous inclusion in the current study.
	Inability or unwillingness of patient or patient’s personal representative to give written informed consent.

CI, Chief Investigator; EVD, External ventricular drain; IL, interleukin; IL-1Ra, interleukin-1 receptor antagonist; SAH, subarachnoid haemorrhage; TNF, tumour necrosis factor.

Informed consent was obtained from the patient or the patient’s representative. Demographic and clinical details were recorded. SAH severity was graded clinically using the World Federation of Neurosurgical Societies (WFNS) score [21] and radiologically using the Fisher grade [22]. The randomisation service was provided by an independent third-party professional company, Sealed Envelope (London, UK; http://www.sealedenvelope.com). After consent the patient was randomised, baseline clinical assessment was performed, and CSF (2 mL) and plasma (5 mL) samples were taken. This was immediately followed by administration of placebo or IL-1Ra. IL-1Ra was injected as Kineret, a recombinant, non-glycosylated form of human IL-1Ra [23]. An IV bolus of 500 mg, given over 1 minute, was immediately followed by a 10 mg/kg/hr infusion over 24 hours. CSF and plasma samples were taken at 6, 12, 24 (end of infusion), 36, 48, and 72 hours. Adverse event reporting was carried out according to Medicines and Healthcare Products Regulatory Agency (MHRA) and sponsor regulations.

### Cytokine assays

Blood samples were collected into tubes containing ethylenediaminetetraacetic acid (EDTA) and centrifuged at 2,000 *g* at 4°C for 15 minutes. Plasma was frozen at −70°C. The CSF was sampled from the patient’s external ventricular drain (EVD), after discarding the first 2 mL, and processed as for plasma. IL-1Ra concentrations were measured by enzyme-linked immunosorbent assay (ELISA), as described elsewhere [19]. Monocyte chemoattractant protein-1 (MCP-1), IL-1β, IL-6, IL-8, IL-10, and tumour necrosis factor-alpha (TNF-α) concentrations in plasma and CSF, and CRP in plasma, were measured using Luminex bead technology (Luminex, Austin, TX, USA). Bio-Plex COOH beads (Bio-Rad Laboratories, Hemel Hempstead, UK) were coupled to Pelikine anti-IL-1β (catalogue number, Cat: M9334) anti-IL-6 (Cat: M191602), anti-IL-8 (Cat: M191802), anti-IL-10 (Cat: M191002), or anti-TNF-α (Cat: M192302) monoclonal antibodies (Mast Group, Bootle, UK), R&D anti-IL-1α (Cat: 840201) or anti-MCP-1 (Cat: 840204) antibodies (R&D Systems, Minneapolis, MN, USA), or Biodesign anti-CRP monoclonal antibody (Biodesign anti-CRP; Cat: M86842M, clone C2), using the Bio-Plex amine coupling kit (Cat: 171–406001). Detection antibodies were Pelikine anti-IL-1β (Cat: M193404), anti-IL-6 (Cat: M191604), anti-IL-8 (Cat: M191804), and anti-TNF-α (Cat: M192304) from Mast Group, anti IL-1α (Cat: M193404) or anti-MCP-1 (Cat: 840205) from R&D Systems. The plasma IL-6, IL-8, IL-10, IL-1β, MCP-1, and TNF-α assays were conducted as a 7-plex, using 15% horse serum, 5% bovine serum, and 1% mouse serum in high performance ELISA buffer (HPE; Pelikine) as a diluent. CSF assays were conducted as a 4-plex (IL-1α, IL-1β, IL-10, and TNF-α) in HPE with 1% horse serum, or a 3-plex (IL-6, IL-8, and MCP-1) in HPE. All standards were calibrated against current National Institute for Biological Standards and Control (NIBSC, South Mimms, UK) standards. Plasma CRP was measured in a single-plex competitive assay, with 10% horse serum, 5% bovine serum, and 1% mouse serum diluent in Tris-buffered saline. The competitor was CRP (P100-0; SCIPAC, Sittingbourne, UK) that had been biotinylated with Pierce EZ-Link Sulfo-NHS-LC-LC-Biotin (Pierce, Rockford, IL, USA). The assay was calibrated with respect to NIBSC human CRP (NIBSC; 85/506). Binding of biotinylated CRP was assessed, following addition of R-phycoerythrin streptavidin (Jackson ImmunoResearch Laboratories Inc, Stratech, Newmarket, UK; Cat: 016-110-084), using a Bio-Plex 200 system. Multiplex assay diluents were matched to plasma or CSF, with three or four quality controls (QCs) for each analyte. The assay performance across the assays in this study is provided in Table 2, in terms of sensitivity and inter-assay coefficient of variation (CV) for these QCs.

**Table 2:** Assay performance

**Analyte**	**Plasma**		**CSF**	
	**Sensitivity (pg/mL)**	**Inter-assay CV range (QC concentration range)**	**Sensitivity (pg/mL)**	**Inter-assay CV range (QC concentration range)**
IL-1Ra	16	8.2 to 3.2 (360 to 10,009 pg/mL)	Same assay as for plasma	
IL-1α	6.1	9.0 to 4.2 (25 to 366 pg/mL)	4.75	6.6 to 9.2 (20 to 800 pg/mL)
IL-1β	1.3	10.3 to 6.3 (17.5 to 146 pg/mL)	0.48	30.3 to 10.9 (1.3 to 10.9 pg/mL)
IL-6	1.7	5.9 to 2 (41 to 322 pg/mL)	17	7.1 to 2.6 (213 to 1,229 pg/mL)
IL-8	4.7	7.9 to 16 (109 to 975 pg/mL)	17	8.9 to 15.8 (185 to 9,152 pg/mL)
IL-10	1.3	10.3 to 6.26 (7.5 to 146 pg/mL)	0.82	10.6 to 5.5 (5.16 to 232 pg/mL)
MCP-1	45	19.5 to 11.3 (207 to 1,759 pg/mL)	147	(19 to 6.5 (9,303 to 11,275 pg/mL)
TNF-α	0.35	6.7 to 7.0 (16.8 to 159 pg/mL)	2.5	25 to 15.8 (4.3 to 28.1 pg/mL)
CRP	350	14.8 to 1 (9.2 to 97 mg/L)	ND	

The assay performance data is only for assays used in this study. The lowest sensitivity of the assays is shown and represents either the non-sample value (+2 SD), or the lowest point on the standard curve. Inter-assay CVs are shown from the lowest to the highest concentration of QCs (three or four) that were included in all assays. CRP, C-reactive protein; CV, coefficient of variation; IL, interleukin; IL-1Ra, interleukin-1 receptor antagonist; MCP-1, monocyte chemoattractant protein-1; ND, not determined; QC, quality control; TNF, tumour necrosis factor.

### Statistical analysis

The primary outcome measure was the area under the curve (AUC) for CSF IL-6 concentration, between 6 and 24 hours from the start of the infusion, adjusted for baseline. Values were log-transformed before analysis and adjustment was achieved by subtraction of the baseline value from values at all time points prior to calculation of AUC. Comparisons between the two treatment groups were made using Student’s *t*-test. Similar analyses were carried out for IL-1α, IL-1β, IL-8, IL-10, MCP-1, TNF-α, and CRP. Statistical analyses were performed with Stata version 12 (StataCorp, College Station, TX, USA). The target sample size was intended to include 16 participants per treatment group, including replacement of participants in whom it was not possible to obtain all samples up to 24 hours from the start of infusion. This sample size gave 80% power at the 5% significance level to detect differences of one standard deviation in outcomes between treatment groups, similar to that seen for ischaemic stroke patients in our previous study [19].

## Results

### Recruitment and baseline characteristics

**Figure 1:**
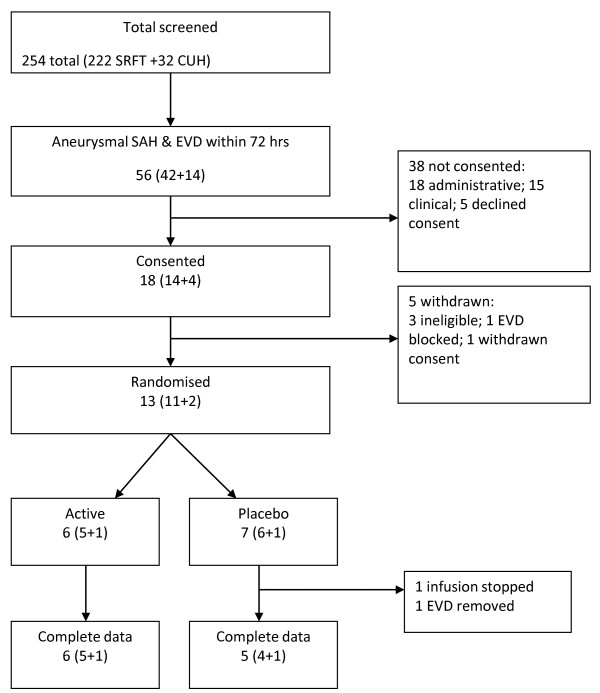
**Flow chart showing the screening and recruitment numbers at Salford Royal Foundation Trust and Addenbrooke’s Hospital.** CUH, Cambridge University Hospitals; EVD, external ventricular drain; SRFT, Salford Royal Foundation Trust.

A total of 254 patients with SAH were admitted to the centres between June 2009 and July 2010. Of these, 56 patients had a confirmed aneurysm and required EVD within 72 hours of ictus. A summary flow chart of screening and recruitment is shown in Figure 1. Overall, 18 patients were recruited into the trial: seven patients received placebo, six patients received IL-1Ra, and five patients were withdrawn from the study prior to randomisation. One patient who received placebo did not complete the 24-hour infusion and analyses were based on the 12 patients in whom the infusion was completed (six placebo, six IL-1Ra). A total of 38 potentially eligible patients were not recruited. Table 3 summarises the baseline characteristics of each treatment group. There were no adverse or serious adverse events attributable to the study drug (Table 4).

**Table 3:** Characteristics of each treatment group

	**All patients (n = 13**^**a**^**)**	**Placebo (n = 7)**	**IL-1Ra (n = 6)**
Mean age (range) (years)	54 (40 to 69)	50 (42 to 61)	58 (40 to 69)
Male/female	3/10	1/6	2/4
WFNS grade			
1	1	1	0
2	5	4	1
3	0	0	0
4	3	1	2
5	4	1	3
Fisher grade			
3	3	2	1
4	10	5	5
Aneurysm location			
Anterior circulation	9	5	4
Posterior circulation	4	2	2

^a^n = 13, as this includes all patients in whom an infusion was started. One patient did not complete the infusion; therefore analyses included only 12 patients. IL-1Ra, interleukin-1 receptor antagonist; WFNS, World Federation of Neurosurgical Societies.

**Table 4:** Adverse and serious adverse events

**Placebo**	**IL-1Ra**
Fluctuating GCS	Raised ICP; hypotension^a^
Desaturation; cardiac arrhythmia; meningitis	Chest sepsis; focal seizures cardiac arrhythmia; increased urine output; increased CRP^a^
IV line infection; chest infection; focal seizure	Ventilator-associated pneumonia
Leaking wound^a^	
Acute agitation^a^; pyrexia of unknown origin^a^	

^a^The 18 adverse and serious adverse events are shown for the five participants that received placebo and the three that received IL-1Ra, in whom they occurred. CRP, C-reactive protein; GCS, Glasgow Coma Scale; ICP, intracranial pressure; IL-1Ra, interleukin-1 receptor antagonist; IV, intravenous.

### Changes in plasma and CSF IL-6 following IL-1Ra administration

**Figure 2:**
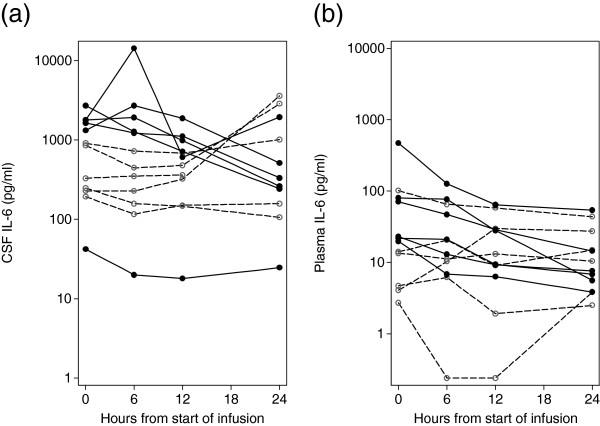
**CSF and plasma IL-6 concentrations for 12 participants over 24 hours from the start of infusion. (a)** CSF IL-6 and **(b)** plasma IL-6 concentrations. Solid symbols and lines represent patients that received IL-1Ra. Open symbols and dashed lines represent patients that received placebo. CSF, cerebrospinal fluid; IL, interleukin; IL-1Ra, interleukin-1 receptor antagonist.

**Figure 3:**
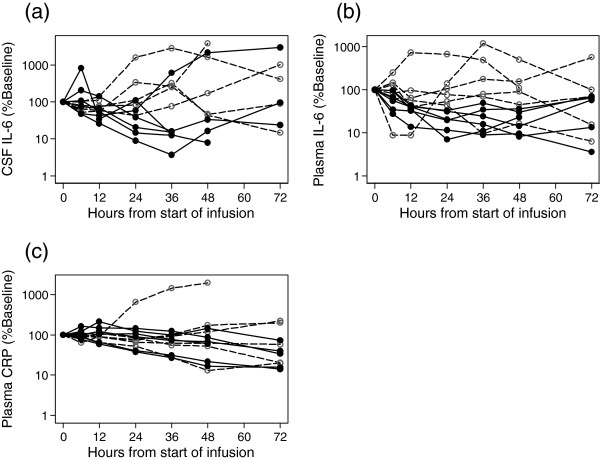
**Change from baseline for CSF IL-6, plasma IL-6, and plasma CRP for 12 participants over 72 hours. (a)** CSF IL-6, **(b)** plasma IL-6, and **(c)** plasma CRP. Solid symbols and lines represent patients that received IL-1Ra. Open symbols and dashed lines represent patients that received placebo. CSF, cerebrospinal fluid; CRP, C-reactive protein; IL, interleukin; IL-1Ra, interleukin-1 receptor antagonist.

The mean CSF IL-6 concentration, prior to infusion was 1,001 pg/mL (range 42 to 2,698 pg/mL). The mean plasma IL-6 concentration in all patients at this time point was 69 pg/mL (range 3 to 469 pg/mL). The CSF IL-6 concentrations reduced over the first 24 hours from the start of infusion in all six of the active group and in three of the six controls (Figure 2a). A similar pattern occurred for plasma IL-6 concentrations, with reductions for five of the six active compared with two of the five control participants (Figure 2b). Samples of CSF were not available for three participants at 48 hours and five participants at 72 hours, and plasma samples were not available for two participants at 72 hours, but the 72-hour CSF and plasma IL-6 kinetics are shown for the remaining participant samples in Figure 3.

### Changes in other measures of inflammation

Plasma and CSF values for IL-1β, IL-1α, and TNF-α were near or below the limits of detection for most samples. Details of AUC from 6 to 24 hours for other outcome measures, both unadjusted (raw) and adjusted for baseline, are given in Table 5. The baseline-adjusted AUC difference for IL-6 in CSF was 12.8, which is essentially a difference of one standard deviation (12.3) for the combined data. This is consistent with the difference anticipated from the power calculation, for the numbers recruited. Similarly, the baseline-adjusted AUC difference for IL-6 in plasma was 18.6, compared with one standard deviation for the combined data (17.2). Reductions in AUC for CSF and plasma IL-8 and IL-10 were also seen, although again the differences were not significant. Plasma CRP was not affected by IL-1Ra over the 6- to 24-hour period, but reduced over the following 24 hours in the IL-Ra cohort, relative to placebo (Figure 3b).

**Table 5:** Analysis of baseline-adjusted AUC from 6 to 24 hours for all outcome measures

		**Placebo**		**IL-1Ra**			
**Mediator**	**Location**	**Raw**	**Adjusted**	**Raw**	**Adjusted**	**Difference**	***P *** **value**
IL-6	CSF	107.3	0.2	111.0	−12.6	12.8 (−2.1 to 27.7)	0.08
	Plasma	37.7	−3.7	49.7	−22.3	18.6 (−0.6 to 37.7)	0.06
IL-8	CSF	114.8	4.8	104.7	−2.3	7.1 (−5.4 to 19.5)	0.23
	Plasma	37.8	6.4	39.8	−0.3	6.7 (−4.9 to 18.3)	0.23
IL-10	CSF	14.6	−6.3	25.0	−8.1	1.8 (−8.6 to 12.2)	0.70
	Plasma	6.7	0.5	9.1	−17.1	17.6 (4.3 to 30.9)	0.01
MCP-1	CSF	139.8	1.5	145.4	−4.0	5.5 (−3.9 to 15.0)	0.22
	Plasma	68.7	−4.3	79.2	−4.0	−0.3 (−14.0 to 13.3)	0.96
CRP	Plasma	65.2	−1.6	84.5	−0.5	−1.1 (−10.3 to 8.1)	0.79

Figures are the mean AUC for the natural logarithm of each marker plotted against time from 6 to 24 hours. Both ‘raw’ (actual values) and baseline-adjusted (subtracting the rectangular area defined by the height of the baseline reading across the 18-hour period) are presented. The mean difference (95% CI) and associated *P* value are given for the adjusted values. AUC, area under the curve; CRP, C-reactive protein; CSF, cerebrospinal fluid; IL, interleukin; IL-1Ra, interleukin-1 receptor antagonist; MCP-1, monocyte chemoattractant protein-1.

### Clinical outcome

Although the study was not designed or powered to measure clinical outcome, the Glasgow Outcome Score (GOS) at 6 months was obtained from the clinical notes and reported for each patient who received placebo or IL-1Ra in the study (Table 6). There was no evidence of difference between treatment groups.

**Table 6:** GOS scores for patients at 6 months after SAH

**Treatment group**	**GOS**	
	**Good (GOS 4 to 5)**	**Poor (GOS 1 to 3)**
Placebo	5	1
IL-1Ra	5	1
Total	10	2

GOS, Glasgow Outcome Score; IL-1Ra, interleukin-1 receptor antagonist; SAH, subarachnoid haemorrhage.

## Discussion

We observed that IV IL-1Ra reduced the concentration of IL-6 in CSF and plasma to the extent predicted, although the numbers of participants it was possible to recruit meant that statistical significance could not be achieved. This provides evidence that peripherally administered IL-1Ra has a biological effect within the central nervous system (CNS) after acute brain injury in humans. It is not certain whether IL-6 itself may be causally involved in contributing to pathology, but both peripheral and central IL-6 have been shown to be associated with outcome [24,25]. However, here we have simply used it as a biological marker to monitor the level of inflammation that results after SAH. Although we were interested in how other markers might respond, we chose IL-6 at the outset as the primary marker, to reduce the difficulty of interpreting *P* values from multiple statistical analyses. As such, the data support the rationale for developing IL-1Ra as a therapy to attenuate the neuroinflammatory response, and possibly the development of DCI, after SAH.

The higher IL-6 concentrations in CSF, compared to plasma, are consistent with previous studies [26], and indicate that changes in CSF do not result from IL-1Ra altering peripheral IL-6 production before translocation to the CNS. We had anticipated a lag in response to IL-1Ra infusion, which is why the analysis is from 6 hours. However, as shown by data for those patients where data was available beyond 24 hours, the effect almost certainly continues beyond this time point. Although sample analysis at later times may demonstrate a greater difference, we are reluctant to attribute significance on the basis of post-hoc analysis, with missing data. Concentrations of CSF and plasma IL-1β and TNF were very low or undetectable, as also found previously [26]. The other cytokines, IL-8, IL-10, and MCP-1 showed differences in the direction expected if IL-1Ra was acting in an anti-inflammatory manner but, as for IL-6, did not reach statistical significance. Consistent with IL-6 as the major driver of the CRP response, plasma CRP concentrations fell in the treatment group, relative to placebo, but only after the primary analysis period.

The study was limited by the small number of patients, mainly due to difficulties of recruiting from a cohort of critically unwell patients. The treatment arm also had patients with a higher WFNS, and started with a higher median IL-6 value. This may have mitigated against observing a treatment effect as more severe implies a bigger drive to counter with therapeutic intervention. The age and gender of patients, and aneurysm location, was however representative of the SAH population as a whole. In keeping with previous clinical studies involving critically unwell patients, IV IL-1Ra was found to be safe and no reported adverse events could be attributed to the drug.

IL-1Ra is unique as a candidate for treatment of the inflammatory response that is induced by SAH and there are extensive preclinical and clinical pharmacokinetic data supporting the role of IL-1Ra as a neuroprotective agent [11]. This contrasts with other candidate treatments in SAH, such as clazosentan and magnesium sulphate (MgSO_4_), where the lack of patient pharmacokinetic and pharmacodynamic data may partly explain the recent failure of phase III clinical trials [27,28].

## Conclusions

Reduction of IL-6 concentration in CSF of patients with SAH indicates that IV administered IL-1Ra may act within the CNS directly or indirectly to attenuate the early inflammatory response to SAH. This suggests it is a promising therapeutic option for the prevention of inflammation and DCI in SAH. Larger trials are required in order to confirm its efficacy and impact on clinical outcome.

## Abbreviations

AUC: Area under the curve: BBB, Blood–brain barrier; CNS: Central nervous system; CRP: C-reactive protein; CSF: Cerebrospinal fluid; CV: Coefficient of variation; DCI: Delayed cerebral ischaemia; EDTA: Ethylenediaminetetraacetic acid; ELISA: Enzyme-linked immunosorbent assay; EVD: External ventricular drain; GOS: Glasgow outcome score; HPE: High performance ELISA; IL: Interleukin; IL-1Ra: Interleukin-1 receptor antagonist; IV: Intravenous; MCP-1: Monocyte chemoattractant protein-1; MHRA: Medicines and healthcare products regulatory agency; NIBSC: National Institute for Biological Standards and Control; QC: Quality control; SAH: Subarachnoid haemorrhage; SC: Subcutaneous; TNF: Tumour necrosis factor; WFNS: World federation of neurosurgical societies.

## Competing interests

The authors declare that they have no competing interests.

## Authors’ contributions

PT, ATK, SJH, NJR, PJH, and AV conceived the study and designed the protocols with NS and SH. Patients’ samples and details were collected by NS, JG, SG, and SH. Samples were analysed by MH and data was analysed and interpreted by AV, NS, SJH, and ATK. The manuscript was drafted by NS and SJH, with input from all other authors, who also approved the final manuscript.
